# Species Diversity, Seasonal Dynamics, and Vertical Distribution of Litter—Dwelling Thrips in an Urban Forest Remnant of South China

**DOI:** 10.1673/031.012.6701

**Published:** 2012-05-24

**Authors:** Jun Wang, Xiaoli Tong

**Affiliations:** ^1^ College of Natural Resources and Environment, South China Agricultural University, Guangzhou 510642, China; ^2^ College of Plant Science, Jilin University, Changchun 130062, China

**Keywords:** insect diversity, leaf—litter thrips, soil invertebrates, spatial and temporal distribution, Thysanoptera

## Abstract

Litter—dwelling thrips are an important component of soil macroinvertebrates in tropical and subtropical regions. However, little is known about assemblage composition, seasonal abundance and vertical distribution of litter—dwelling thrips. A survey of forest litter—dwelling thrips and other soil macroinvertebrates was conducted in an urban forest remnant at Guangzhou, China during 2004–2005 and 2008–2009. A total of 835 Tullgren samples were collected during the study. Thysanoptera constituted 6.5% of total litter—dwelling macroinvertebrate individuals extracted, representing three families, 19 genera, and 25 species. *Psalidothrips ascitus* Ananthakrishnan (Thysanoptera: Phlaeothripidae) and *Hyidiothrips guangdongensis* Wang, Tong and Zhang represented 78.5% of all individuals of litter—dwelling thrips during the survey. Numbers of species and density of leaf—litter thrips fluctuated between different months. Density of litter thrips increased from March until October, reaching a maximum of 41.1 individuals/m2 followed by a decrease. In January and February only a few larval thrips were present. Species diversity gradually increased from July (four species) to December (10 species), and then declined rapidly. The vertical distribution showed that the leaf—litter thrips species richness and abundance decreased significantly with soil depth; they were found only in the litter layer and upper soil layer (0-5 cm in depth) and were entirely absent in deeper soil layers. The results suggest that litter—dwelling thrips are a common group of litter invertebrates with high species diversity in subtropical regions. These urban forest remnants should be given special consideration in forest conservation planning, because of their significance as refugia for the litter invertebrate assemblages, especially for leaf—litter thrips.

## Introduction

Urban forest remnants perform a varied range of useful functions and services, such as refuges for native biodiversity, wildlife corridors, and opportunities for understanding and appreciating biodiversity (Burns et al. 2000). The diversity of butterflies, beetles, and spiders has been reported to be rich in urban forest remnants (McGeoch and Chown 1997; Bolger et al. 2000; Alaruikka et al. 2002; Brown and Freitas 2002; Gibb and Hochuli 2002). But litter invertebrates of urban forest remnants are poorly understood because of their generally small size and cryptic habits. However, litter invertebrates are of great importance for nutrient recycling (Hattenschwiler et al. 2005). Also, they contribute to studies of comparative biological diversity, forest management, and conservation, and can act as bioindicators of ecological sustainability (Nakamura et al. 2003). Furthermore, litter invertebrates are a major link in food webs (Anderson 2000).

Insects of the order Thysanoptera constitute one of most common and important components of the soil invertebrate fauna. This group includes approximately 6000 described species worldwide, classified into nine families (Mound 2011), with at least 2500 species found in forest litter where they feed on the fungi associated with the early stages of leaf decay (Mound 2005). Litter— dwelling thrips are particularly diverse in the subtropics and tropics, with up to 50% of thysanopteran species in these regions (Mound 2002). For example, almost 50 species of leaf—litter thrips were described from a single area of forest 50 km in diameter in southern Brazil (Mound 1977). In subtropical China, litter—dwelling thrips also have a high relative abundance among litter macroinvertebrates (Li et al. 2004a, 2004b; Wang et al. 2008). The diversity of these leaf— litter thrips is usually related to environmental factors, including temperature and humidity of the soil, the plant species that produce the litter, and the species of fungi involved in decomposition (Ananthakrishnan 1996). These litter-dwelling thrips can be a potential indicator to assess changes in the forest environment (Mound 1977; Ananthakrishnan 1996). However, many aspects of assemblage composition, seasonal abundance, and vertical distribution are poorly known for litter— colonizing thrips.

To determine the value of urban forest remnants as suitable habitat and reservoirs for species of litter invertebrates, including litter— dwelling thrips, and to identify the threats to their conservation, a better understanding of litter invertebrate composition is required. The biodiversity values of urban forest remnants were evaluated by examining seasonal fluctuations and vertical distribution of litter— dwelling invertebrates, with particular emphasis on thrips, in an urban forest remnant of subtropical China.

## Materials and Methods

### Study area

**Figure 1.**
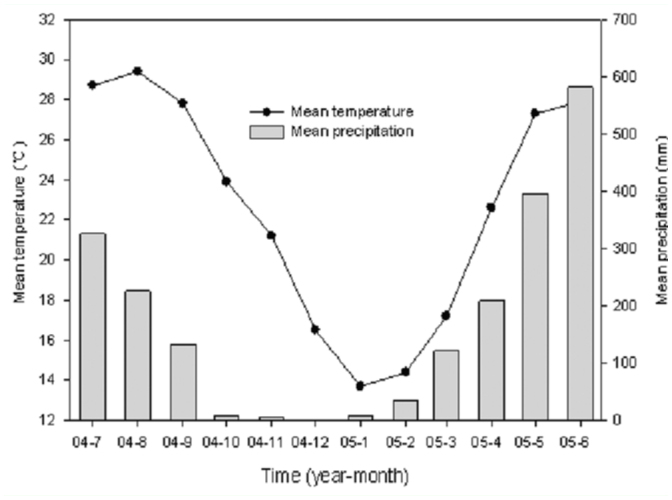
Seasonal change in monthly mean air temperature (solid circles) and precipitation (shaded bars) during the study period in an urban forest remnant at Guangzhou, July 2004 to June 2005. High quality figures are available online.

This study was conducted on the Changgangshan Nature Reserve (23° 09′ 20″ 23° 09′ 35″ N, 113° 21′ 08″ - 113° 21′ 26″ E), a subtropical urban forest remnant that is located on the campus of South China Agricultural University, Guangzhou, China. This remnant is surrounded on three sides by campus buildings, and on the fourth side by a motorway and farmland. The overall size of the reserve is 15 ha. Mean monthly temperatures range from 13.7 ^°^C in January to 29.4 ^°^C in August; mean annual precipitation was about 2050 mm and occurs mainly between April and September (Figure 1).

The vegetation of the remnant would have been subtropical monsoonal rainforest, but the original forests have been largely destroyed. Most of the landscape has been transformed to secondary forest, shrub land, and plantation of *Eucalyptus* and *Acacia.* However, in 1972 an arboretum was established in the reserve as an ex—situ conservation area. Currently 1200 vascular plant species representing 171 families and 608 genera are recorded in the arboretum (Wu and Feng 2006). The survey sites in this study were located predominantly in the secondary forest. The dominant canopy tree species were *Schima superba* (Ericales: Theaceae), *Castanopsis fissa* (Fagales: Fagaceae), *Castanopsis kawakamii*, *Vatica mangachapoi* (Malvales: Dipterocarpaceae), and *Hopea hainanensis.* These trees were up to 10–15 m in height and 20–40 cm dbh. The forest understory was dense, consisting of herbs and grasses. The thickness of the leaf litter layer varied considerably, but was generally in the range of 1–3 cm.

### Sampling: seasonal fluctuation

Species diversity and seasonal abundance of litter thrips and other litter macroinvertebrates were quantitatively sampled monthly from July 2004 to June 2005. In each sampling, five plots (10 × 10 m) were randomly selected, and the distance between each sample plot was more than 50 m; five quadrate litter samples (50 × 50 cm) were selected in each plot, four in each corner and one in the center. A total of 275 litter samples were then placed in labeled plastic bags and extracted by means of a modified Tullgren funnel in lab.

### Sampling: vertical distribution

The vertical distribution of litter—dwelling thrips and other litter and soil macro– invertebrates was quantitatively sampled every three months from April 2008 to January 2009 (four sampling events). In each sampling, seven plots (10 × 10 m) were randomly selected and the distance between each sample plot was more than 50 m; five quadrate samples (10 × 10 cm) were selected in each plot, four in each corner and one in the center. Each quadrate sample consisted of the litter layer and a soil core measuring 0.01 m (10 × 10 cm) and 15 cm deep, and divided into four layers: the litter layer, upper soil layer (0–5 cm in depth), middle soil layer (5– 10 cm), and lower soil layer (10–15 cm). A total of 560 litter or soil samples were then placed in labeled plastic bags and the macro– invertebrates from each layer were extracted by the modified Tullgren funnel. Each sampling event in the field was completed within six hours to minimize phenological changes.

### Sorting and identification

Litter—dwelling thrips and other soil macroinvertebrates were extracted with the modified Tullgren funnels using 60 W bulbs suspended 10 cm above the top of the samples over 10 hours until the litter was dry and fragile; individuals were then preserved in 75% alcohol. All extractions were completed within six days. The macro—invertebrate samples were sorted into order level and counted under a dissecting microscope, but the adults of leaf—litter thrips were identified to the species level, and the larval stages of thrips were separated under the category of “thrips larvae” and total numbers were documented.

### Data analysis

Species richness, density (individuals/m2), relative abundance, and frequency were applied to indicate the diversity of litter— dwelling thrips. Relative abundance refers to the total number of specimens for a particular species divided by the total number of all litter thrips, while frequency expresses the number of individuals of a species collected in a month divided by the total number of months. A “dominant group” is defined as having a relative abundance of more than 10%; the relative abundance of “ordinary groups” is between 1% and 10%; “rare group” is less than 1% relative abundance. The density of each thrips species in each month was the mean of 25 quadrate samples from five plots, presented as mean ± SE. The effects of the four horizons (litter layer, 0–5, 5–10, 10–15 cm) on densities of soil invertebrate taxa was determined by parametric ANOVA with pairwise comparisons using Tukey HSD. Prior to analyses, densities (x) were log(x+1)transformed to improve homogeneity of variances. The analyses were carried out using SPSS version 12.0.

**Table 1.**
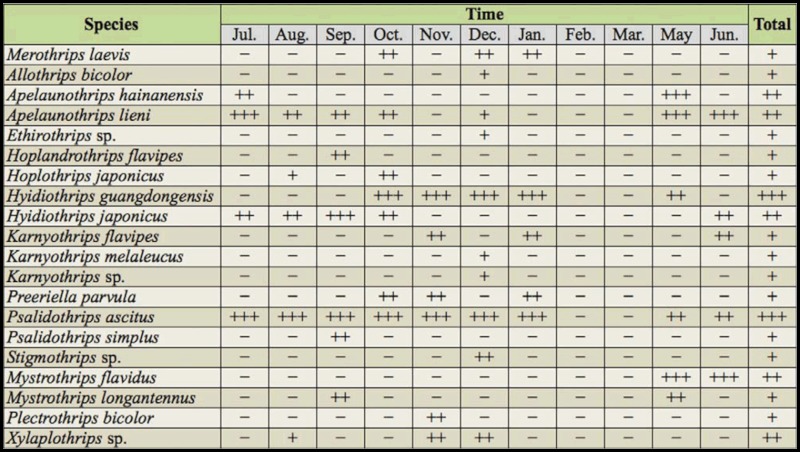
Species composition and relative abundance of litter—dwelling thrips in leaf litter in an urban forest remnant at Guangzhou, China (July 2004 to June 2005).

## Results

### Seasonal fluctuation

A total of 21,817 litter invertebrates were collected, belonging to 22 groups in nine classes and three phyla. Acarina and Collembola accounted for 55.7% and 14.7% of the total individuals of litter invertebrates, respectively, and were considered to be “the dominant groups”. The individuals of the ordinary groups including Helminthomorpha, Araneae, Isopoda, Thysanoptera, Coleoptera, Hymenoptera, Lepidoptera larvae, and Diptera larvae together occupied 27.0% of the total. Thysanoptera were the third most abundant group, accounting for 6.5% of total individuals. Araneae, Acarina, Collembola, Thysanoptera, Coleoptera, Lepidoptera larvae, and Hymenoptera were the most frequent groups; these were collected in every month. Mean density of litter thrips was 20.6 ± 15.6 individuals/m2. Altogether, 1024 thrips adults were captured, representing 25 species in 19 genera and three families.

**Figure 2.**
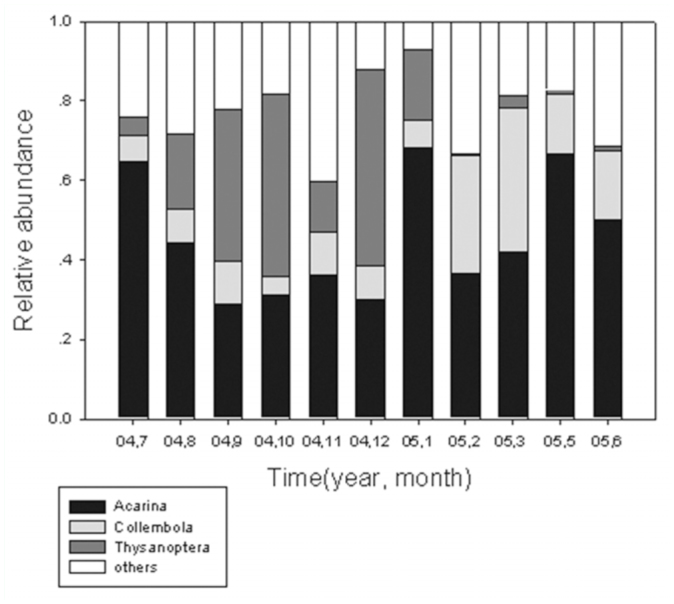
Seasonal fluctuation of relative abundance of litter— inhabiting Acarina, Collembola, and Thysanoptera in an urban forest remnant at Guangzhou, China (July 2004 to June 2005). High quality figures are available online.

Relative abundance of these main groups fluctuated distinctly between months (Figure 2). Thysanoptera, for example, was a dominant group in the litter invertebrate assemblage from August to January, but was a rare group in February. In December, 253 individuals of litter thrips represented 49.3% of the litter invertebrates, and this was the maximum relative abundance for the year. The minimum relative abundance was recorded in February (0.4%).

In total, 20 species of litter—dwelling adult thrips (997 individuals), representing 14 genera and two families, were collected (Table 1). Most species (19) and genera (13) belonged to the family Phlaeothripidae, with one species from the family Merothripidae (*Merothrips laevis* Hood). *Psalidothrips ascitus* Ananthakrishnan and *Hyidiothrips guangdongensis* Wang, Tong, and Zhang were the most abundant species, accounting for 55.0% and 23.5%, respectively. The remaining species, including *Apelaunothrips lieni* Okajima, *Apelaunothrips hainanensis* Zhang and Tong, *Hyidiothrips japonicas* Okajima, *Mystrothrips flavidus* Okajima, and *Xylaplothrips* sp., accounted for 17.7% of the litter—dwelling thrips individuals. In addition to litter—dwelling thrips, 27 individuals of the family Thripidae were collected, representing five species: *Dendrothrips minowai* Priesner, *Scirtothrips dorsalis* Hood, *Hydatothrips aureus* Bhatti, *Phibalothrips peringueyi* (Faure) and *Selenothrips rubrocinctus* (Giard). These thripids are flower—living or leaf— feeding and only enter the litter or soil to pupate.

There was an effect of sampling sites and sampling months on the richness and abundance of litter—dwelling thrips. The highest number of individuals (89) and the highest species richness (five species) in a sample both were recorded in December. Species composition also varied among different months. The number of species gradually increased from July to December, and the richness was highest in December (10 species) (Table 1). No thrips adults were collected in February and March in the study area, but thrips larvae were found in every month.

**Figure 3.**
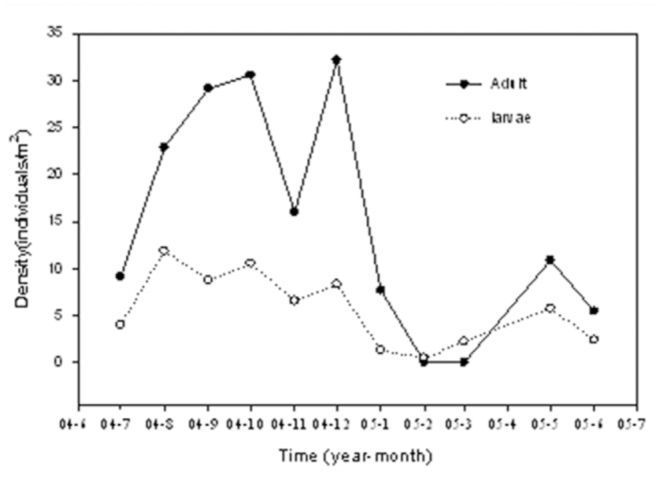
Seasonal abundance (mean density) of litter—dwelling thrips adult and larvae in an urban forest remnant of Guangzhou, China (July 2004 to June 2005). High quality figures are available online.

**Figure 4.**
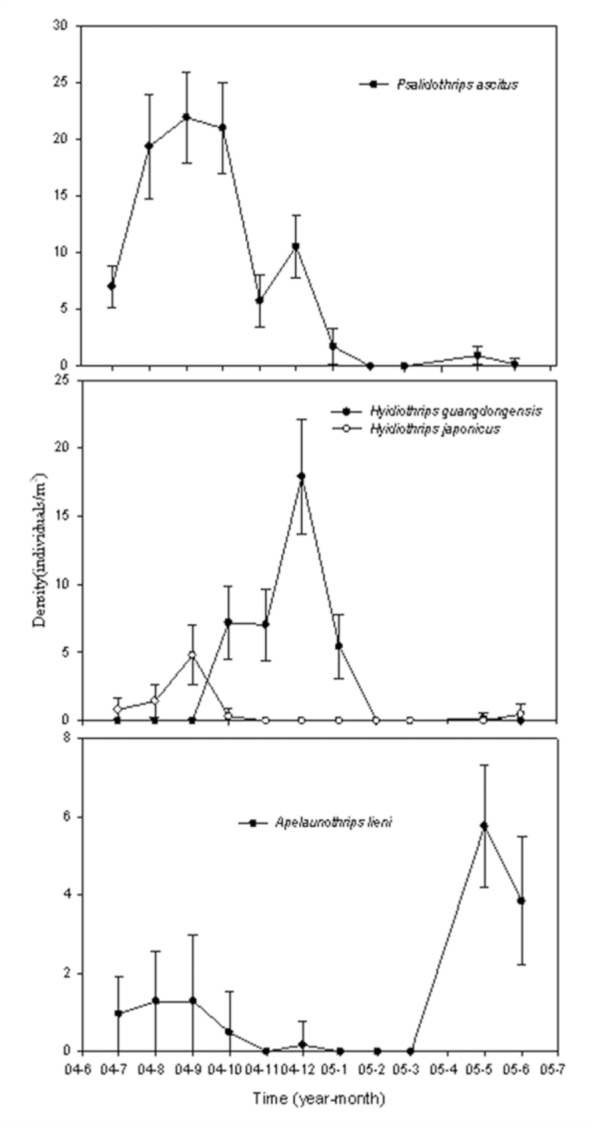
Seasonal abundance (mean density with standard errors) of the four dominant species in an urban forest remnant at Guangzhou, China (July 2004 to June 2005). High quality figures are available online.

Density of litter thrips increased from March until October, reaching a maximum of 41.1 individuals/m2, followed by a subsequent decrease. In the Guangzhou area, 90% of the total annual precipitation was concentrated between April and September (Figure 1), but inconsistent patterns were found in the abundance of litter—dwelling thrips. The density of litter—dwelling thrips adults or larvae fluctuated in different months, the peak of adult density appeared about two months later than that of larvae (Figure 3). Population densities of the major species also varied among months (Figure 4). The density of *P. ascitus* increased from July to September but then decreased gradually. The density of *A. lieni* showed a consistent pattern with that of rainfall with the highest density recorded in May and June. In contrast, the density of *H. guangdongensis* was higher from October to January, in response to the dry seasons. The changes in population density of the two species of *Hyidiothrips* were interesting because they peaked in different months (Figure 4).

### Vertical distribution

A total of 17,503 individual soil animals were collected, belonging to 27 taxa in 11 classes and three phyla. The results of vertical distributions showed that density and taxa number decreased with increasing soil depth. The order of density in the four soil layers follows: leaf litter > surface soil > middle soil > bottom soil; and taxa number follows: surface soil > leaf litter > middle soil > bottom soil. The vertical distribution patterns of soil animals were different between seasons, and the variations were mainly in leaf litter and surface soil layer. In spring, the density was highest in the leaf litter layer, but the taxa number was highest in surface soil layer. The soil animals were likely to live in the litter layer in the summer. Both taxa number and density were highest in the surface soil layer in autumn and winter.

**Figure 5.**
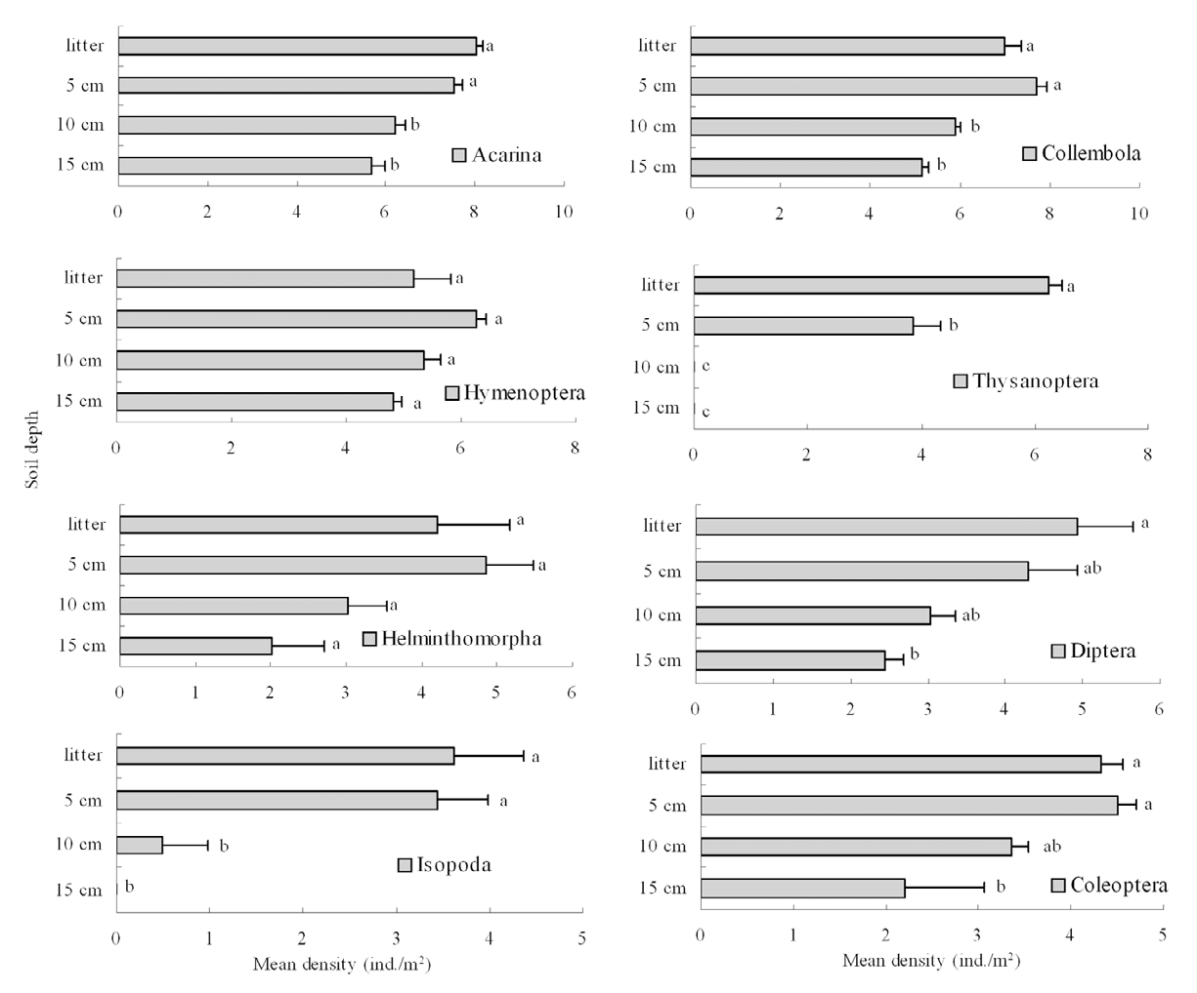
Vertical distribution on mean density of litter—dwelling and other soil macro-invertebrate in the Changgangshan Natural Reserve from April 2008 to January 2009. The same letters meant no significant difference at 0.05 levels of probability (ANOVA, Tukey HSD) and bars with standard errors represent log(x+1)-transformed data. High quality figures are available online.

The vertical distribution patterns of Acarina, Collembola, Helminthomorpha, Hymenoptera, Coleoptera, and Diptera were similar, with the exception of Isopoda and Thysanoptera, which were only found in the litter layer and top soil layer (Figure 5). For thrips, a total of 764 individuals were collected representing 11 species. Table 2 shows that the mean density of litter-dwelling thrips decreased (ANOVA, Tukey HSD, *p* < 0.05) from the litter layer to the soil layers. These species were superficially distributed in the litter and upper soil layers, with no individuals being found in the middle and deeper soil layers.

## Discussion

The thrips extracted from litter consisted of three groups: inhabitants of dead branches or decayed tree, mainly spore—feeding species of the subfamily Idolothripinae (Mound and Palmer 1983); inhabitants of forest litter feeding on fungi associated with the early stages of leaf decay, from the subfamily Phlaeothripinae and the family Merothripidae; and species of the family Thripidae that feed on flowers or green leaves, and only enter the litter accidentally or to pupate. The first two of these three groups can be designated jointly by the name fungus—feeding thrips. However, there were no previous quantitative studies of species diversity and ecology of litter thrips until Thysanoptera were collected from leaf litter while studying the litter invertebrate fauna in southern China. Thrips individuals accounted for 1.4-13.3% of all litter invertebrate individuals in samples from natural forest and different plantation forests of Shimentai Nature Reserve (Li et al. 2004a), 2.3-3.0% in the Nankunshan Nature Reserve (Li et al. 2004b), and 5.0% in the natural forest of Lianhuashan Forest Park, Haifeng (Wang et al. 2008). These studies showed that litter thrips constituted 6.5% of the total litter macro—invertebrate individuals extracted, and that litter thrips are a common group of litter invertebrates with high species diversity and relative abundance in subtropical China.

**Table 2.**
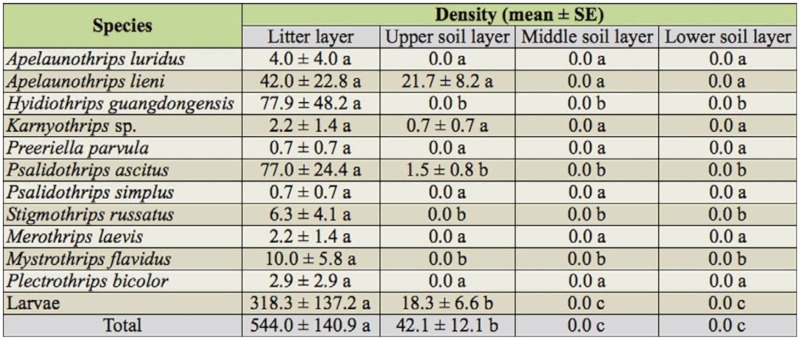
Vertical distribution of mean density (individuals/m^2^) of litter—dwelling thrips in the Changgangshan Natural Reserve (April 2008 to January 2009).

A total of six distribution patterns can be recognized among the 14 genera of litter— dwelling thrips in Changgangshan Nature Reserve. Species with the pantropical distribution are most abundant, accounting for 41.7% of the total genera, including the following five: *Merothrips*, *Apelaunothrips*, *Karnyothrips*, *Preeriella*, and *Plectrothrips*; these genera are found in the tropical and subtropical areas of Asia, Africa, and the Americas. *Stigmothrips* and *Mystrothrips* are distributed across tropical Asia, being found in Japan, Korea, and the India—Malaya area. *Hyidiothrips* and *Psalidothrips* occur in the warmer areas of Asia and the Americas. Excluding the genera with cosmopolitan distributions (*Allothrips* and *Ethirothrips*), 75.0% of litter—dwelling genera are found in tropical and subtropical areas. These Zoogeographie analyses indicate that litter— dwelling thrips in Changgangshan Nature Reserve possess tropical and subtropical characteristics. Litter—dwelling thrips inhabiting forest litter usually have weak flight ability (some species are even wingless), but individual genera or even species are sometimes found in two disconnected continents. Mound (1970) pointed out that certain litter—dwelling species have been moved around the world in hay or straw, or in the ballast of sailing ships. Okajima (1994, 2006) added that parthenogenesis can be important in facilitating the widespread distribution of some species. Our study area was redeveloped as an arboretum following 1972, involving the introduction of some rare and endangered plants, as well as plants offered key national protection. In this replanting process, quantities of litter, soil, and plant debris were also transported from their original sites, along with many associated litter—dwelling thrips.

The individual number and density of litter— dwelling thrips fluctuated among different months, in relation to their life histories and environmental factors, i.e., precipitation and temperature. Temperature and humidity, either independently or in combination, appeared to trigger reproductive polymorphism and the number of eggs of certain litter—dwelling thrips (Ananthakrishnan 1990). In the Guangzhou area, rainfall between April and September accounted for more than 90% of the total annual precipitation (Figure 1). The density and number of species of litter—dwelling thrips were highest in October and December, and the majority of larvae were collected in February and March. Our results suggested that the decrease of abundance and diversity of litter thrips was affected not only by heavy rainfall in monsoonal seasons, but also by drought and low temperature in winter. A similar conclusion was made in Panama (Levings and Windsor 1982).

Each soil animal group generally has a specific pattern of vertical distribution, which indicates that the vertical distribution of soil macroarthropods is important in understanding the interrelationship between the surface litter and deeper soil layers (Ponge 2000). Our study illustrated the occurrence of a gradient in the vertical distribution of soil macroinvertebrate density in a subtropical urban forest remnant, with a few relevant groups inhabiting both litter and soil layers, acting as connectors between both habitats. As illustrated in Figure 5, the pattern of vertical distribution of most soil macroinvertebrates was similar with other studies (Kühnelt 1963; Rogers and Kitching 1998; Roisin et al. 2006). However, in contrast to our expectations, Helminthomorpha was found in deeper soil layers in our study. A total of 11 species of litter-dwelling thrips were found in this vertical distribution study (Table 2); however, only *A. lieni*, *Karnyothrips* sp., *P. ascitus*, and larvae were found inhabiting both litter and the top soil layer (0–5cm), and the rest were superficially distributed only in the litter layer, suggesting that the hyphal mass is more easily accessible to leaf—litter thrips in the litter (Mound 1977). As expected, the biomass and diversity of fungal communities tended to decrease with increasing depth (Jumpponen et al. 2010).

In general, a forest remnant is a patch of native forest around which most or all of the original vegetation has been removed (Burns et al. 2000). However, such remnants can perform a wide range of valued functions and services, acting as refuges for native biodiversity and/or wildlife corridors, as well as providing opportunities for understanding and appreciating biodiversity, etc. Remnants in urban areas are particularly important, because they may be the only natural areas in the urban setting, providing unique opportunities for people living in an urban setting to interact with nature. However, the importance of urban forest remnants has not been acknowledged fully by citizens. Some areas are deforested and replaced with profitable monoculture plantations, and other areas suffer from lack of management practice. In this study, 25 species of leaf litter thrips were recorded in the mini urban forest remnant; two species were new to science, *H. guangdongensis* (Wang et al. 2006) and *Mystrothrips longantennus* (Wang et al. 2008), and two were new records for China, *M. flavidus* and *Plectrothrips bicolor* Okajima. The present study has shown that species diversity and abundance was high in this urban forest remnant, and such small remnants can provide habitats for a significant proportion of thrips fauna occurring in natural forests. Small forest remnants therefore have considerable potential as reservoirs of thrips diversity in highly modified landscapes. The contribution of urban forest remnants to the local sustainability of thrips assemblages emphasizes the importance of maintaining forest remnants in and around cities.

## References

[bibr01] Alaruikka D, Kotze DJ, Mateveinen K, Niemela J. (2002). Carabid beetle and spider assemblages along a forested
urban—rural gradient in Southern Finland.. *Journal of Insect Conservation*.

[bibr02] Ananthakrishnan TN. (1990). *Reproductive
of Biology of Thrips.*.

[bibr03] Ananthakrishnan TN. (1996). *Forest
Litter Insect Communities*, *Biology*,
*and Chemical
Ecology.*.

[bibr04] Anderson JM., Coleman DC, Hendrix PF (2000). Food
web functioning and ecosystem processes: problems and perceptions of
scaling.. *Invertebrates as
Webmasters in
Ecosystems.*.

[bibr05] Bolger DT, Suarez AV, Crooks KR, Morrison SA, Sase TJ. (2000). Arthropods in urban habitat fragmentation in Southern California:
area, age, and edge effects.. *Ecological Applications*.

[bibr06] Brown KS, Freitas AVL. (2002). Butterfly communities of urban forest fragments in Campinas, Sao
Paulo, Brazil: structure, instability, environmental correlates, and
conservation.. *Journal of Insect Conservation*.

[bibr07] Burns B, Barker GM, Harris RJ, Innes J., Saunders D, Craig JL, Mitchell ND (2000). Conifers
and cows: forest survival in New Zealand dairy
landscape.. *Nature
Conservation* 5: *Nature Conservation in Production
Environments*.

[bibr08] Gibb H, Hochuli DF. (2002). Habitat fragmentation in an urban environment: large and small
fragments support different arthropod assemblages.. *Biological Conservation*.

[bibr09] Hattenschwiler S, Tiunov AV, Scheu S. (2005). Biodiversity and litter decomposition in terrestrial
ecosystems.. *Annual Review of Ecology Evolution and Systematics*
3.

[bibr10] Jumpponen A. (2010). Vertical distribution of fungal communities in tallgrass prairie
soil.. *Mycologia*.

[bibr11] Kühnelt W. (1963). Soil—inhabiting arthropoda.. *Annual Review of Entomology*.

[bibr12] Levings SC, Windsor DM., Leigh EG, Rand AS, Windsor DM (1982). Seasonal
and annual variation in litter arthropod
populations.. *The Ecology of a
Tropical Forest Seasonal Rhythms and Longterm
Changes*..

[bibr13] Li ZW, Tong XL, Zhang WQ, Xie GZ, Dai KY. (2004a). Diversity of soil invertebrate assemblages in the forest of
Shimentai Nature Reserve, Guangdong Province.. *Journal of South China Agricultural University*.

[bibr14] Li ZW, Tong XL, Zhang WQ, Xing W, Chen QR. (2004b). Diversity
of soil invertebrate assemblages in the natural and plantation forests of
the Nankunshan Nature Reserve of Guangdong
Province.. Biodiversity
Committee of the Chinese Academy of Sciences, Editor. *Advances in
Biodiversity Conservation and Research in
China*.

[bibr15] McGeoch MA, Chown SL. (1997). Impact of urbanization on a gall–inhabiting Lepidoptera
assemblage: the importance of reserves in urban areas.. *Biodiversity Conservation*.

[bibr16] Mound LA. (1970). Thysanoptera from the Solomon Island.. *Bulletin of the British Museum of Natural History*,
*Entomology Series*.

[bibr17] Mound LA. (1977). Species diversity and the systematics of some New World leaf
litter Thysanoptera (Phlaeothripinae; Glyptothripini).. *Systematic Entomology*.

[bibr18] Mound LA. (2002). Thysanoptera biodiversity in the Neotropics.. *Revista Biologia Tropical*.

[bibr19] Mound LA. (2005). Thysanoptera - Diversity and Interactions.. *Annual Review of Entomology*.

[bibr20] Mound LA. 2011 *Thysanoptera (Thrips) of the World — a checklist.* Available online, www.ento.csiro.au/thysanoptera/worldthrips.html

[bibr21] Mound LA, Palmer JM. (1983). The generic and tribal classification of sporewfeeding
Thysanoptera (Phlaeothripidae: Idolothripinae).. *Bulletin of the British Museum of Natural History*,
*Entomology Series*.

[bibr22] Nakamura A, Proctor H, Catterall CP. (2003). Using soil and litter arthropods to assess the state of
rainforest restoration.. *Ecological Management and Restoration*.

[bibr23] Okajima S. (1994). Habitats and distributions of the Japanese Urothripine species
(Thyanoptera, Phlaeothripidae).. *Japanese Journal of Entomology*.

[bibr24] Okajima S. (2006). *The
Insects of Japan. Volume 2. The suborder Tubulifera
(Thysanoptera).*.

[bibr25] Ponge JF. (2000). Vertical distribution of Collembola (Hexapoda) and their food
resources in organic horizons of beech forests.. *Biology and Fertility of Soils*.

[bibr26] Roisin Y, Dejean A, Corbara B, Orivel J, Samaniego M, Leponce M. (2006). Vertical stratification of the termite assemblage in a
neotropical rainforest.. *Oecologia*.

[bibr27] Rogers DJ, Kitching RL. (1998). Vertical stratification of rainforest collembolan (Collembola:
Insecta) assemblages: description of ecological patterns and hypotheses
concerning their generation.. *Ecography*.

[bibr28] Wang J, Liao QS, Ding WM, Tong XL. (2008). Invertebrate biodiversity in litter layers of natural forest and
*Eucalyptus* plantation in eastern Guangdong,
China.. *Chinese Journal of Applied Ecology*.

[bibr29] Wang J, Tong XL, Zhang WQ. (2006). A new species of the genus *Hyidiothrips*
(Thysanoptera: Phlaeothripidae) from China.. *Zootaxa*.

[bibr30] Wang J, Tong XL, Zhang WQ. (2008). A new species of the genus *Mystrothrips*
(Thysanoptera, Phlaeothripidae) from China.. *Entomological New s*.

[bibr31] Wu YB, Feng ZJ. (2006). Rare and endangered plants and national key protected plants for
ex situ conservation in South China Agricultural University,
Arboretum.. *Journal of South China Agricultural University*.

